# Principal Components Analysis Relates Hippocampal Trisynaptic Circuit to Vividness of Visual Mental Imagery

**DOI:** 10.1002/brb3.71666

**Published:** 2026-08-30

**Authors:** David F. Marks

**Affiliations:** ^1^ Research National Coalition of Independent Scholars South Bend Indiana USA

**Keywords:** hippocampal subfields, principal components analysis, parasubiculum, trisynaptic circuit, visual mental imagery, VVIQ

## Abstract

**Purpose:**

The hippocampal formation serves fundamental processes to support scene construction, learning, memory, and imagination. Here, we use principal components analysis (PCA) to investigate the relation of the trisynaptic circuit, and other hippocampal zones, to individual differences in visual mental imagery ability.

**Method:**

The data from 53 healthy, middle‐ to older‐aged adults with MRI scans of 36 hippocampal subfield volumes were entered into a PCA, which extracted information about individual differences in subfield volumes and their support, or not, of visual mental imagery ability.

**Findings:**

A seven‐factor solution explained 85% of the variance in hippocampal subfield volumes. The first principal component explained 37% of the variance in referencing trisynaptic circuit subfields. The subjects’ ability to form vivid visual mental imagery was indexed with the Vividness of Visual Imagery Questionnaire (VVIQ), which we used to compare the component scores of the 10 highest scoring VVIQ subjects to those of the 10 lowest scoring VVIQ subjects. The high VVIQ group possessed significantly larger component scores than the low VVIQ subjects, with the component referencing the trisynaptic pathway yielding significant differences between the high and low imagery groups. The second principal component indexed a previously unidentified hippocampal region, which referenced four neighboring subfields: the molecular layer, hippocampal tail, presubiculum, and subiculum. All four subfields had strong loadings of between 0.625 and 0.821 on Component 2, which appears to have pinpointed a cohesive structure performing as an integrated network.

**Conclusion:**

These findings suggest that the trisynaptic circuit is critical for the formation of vivid visual imagery with a lesser role for the parasubiculum, while another site consisting of four neighboring subfields (the molecular layer, hippocampal tail, presubiculum, and subiculum) serves alternative functions yet to be established.

## Graphical abstract

Strong associations exist between vividness of conscious visual mental imagery and the trisynaptic circuit and, to a lesser extent, the parasubiculum. A large but previously‐unidentified subregion comprising 49% of the hippocampal formation has no association with the vividness of conscious visual mental imagery. The function of this “quiet” hippocampal region needs to be identified.

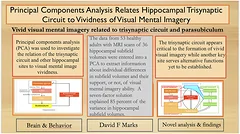

## 1 Introduction

The hippocampal formation serves fundamental processes of scene construction, learning, memory, and imagination. The construction of scenes and memories without this universal mammalian system for memory and mental map production would be inconceivable. The hippocampal formation is associated with two notable circuits, the trisynaptic circuit (TSC) and the Papez circuit (Papez 1937). Of particular interest here is the trisynaptic circuit connecting the perforant pathway with the dentate gyrus (DG), CA3, and CA1. As the largest of the 12 hippocampal subfields, CA1 serves as the target destination for the TSC, operating, in effect, as a hippocampal “engine room.” Here, we focus on the structure and function of the transsynaptic pathway, which goes between the entorhinal cortex layer II to the DG and cornu ammonis (CA) (Lorente de Nó 1934; Ramón y Cajal 1995). Our goal is to examine the degree of involvement of the TSC in its construction of scenes in episodic and declarative memory with the use of vivid, voluntary, visual mental imagery (VMI). Somewhat unexpectedly, we also find a region occupying close to half of the hippocampus that, apparently, plays no role in conscious scene construction.

Individual differences in VMI vividness have been investigated since the pioneering research by Gustav Fechner (Fechner 1860; Betts 1909; Sheehan and Neisser 1969; McKelvie 1995; Marks 1973, 1995, 2023a, 2023b, 2025). The relationships of different subregions of the hippocampal formation with spatial and episodic memory have provided a fruitful avenue for VMI and memory research (e.g., O'Keefe and Nadel 1978; Burgess et al. 2002; Schacter et al. 2012; Maguire and Mullally 2013; Rolls 2013; Moscovitch et al. 2016). Subfields within the TSC—the DG, CA4, CA2/3, and CA1—have all been implicated in pattern separation and completion (Rolls 2013). The DG shows pattern separation ability for discriminating similar stimuli (Baker et al. 2016; Berron et al. 2016), and the CA3 supports pattern separation and/or completion (Bakker et al. 2008; Lacy et al. 2011; Bonnici et al. 2012; Deuker et al. 2014;  Dimsdale‐Zucker et al. 2018; Grande et al. 2019; Hanert et al. 2019;  Molitor et al. 2021). The subiculum supports the ability to separate similar memories (Molitor et al. 2021). The hippocampus appears able to track change by detecting boundaries separating one context, or episode, from another to solve the equivalence problem using shifting representational scales along its longitudinal axis (Maurer and Nadel 2021).

The hippocampal formation shows multiple capabilities beyond learning and memory including (but not only): complex processing of sensory stimuli, even in the unconscious state (Katlowitz et al. 2025), inductive reasoning (Pudhiyidath et al. 2022), recognition of abstract concepts and words (Kafkas et al. 2024), abstraction (Bernardi et al. 2020; Courellis et al. 2024), nonspatial associations (Aminoff et al. 2007), transitive inference (Wendelken and Bunge 2010), and mathematical logic (Friedrich and Friederici 2013; Li et al. 2025). The subregions and organization involved in these activities are not established in great detail, but the hippocampal support of them is unequivocal.

In research on VMI vividness, significant correlations have been reported between hippocampal volumes in areas CA1, CA3, and CA4 with Vividness of Visual Imagery Questionnaire (VVIQ) (Tabi et al. 2022). People with high VVIQ scores also have been shown to have larger hippocampal subfields and stronger visual short‐term memory than people with low VVIQ scores (Marks 2025). Here, we use PCA to investigate the latent structure of hippocampal subfield volume space to reveal principal components and examine their associations with vividness of VMI.

**FIGURE 1:**
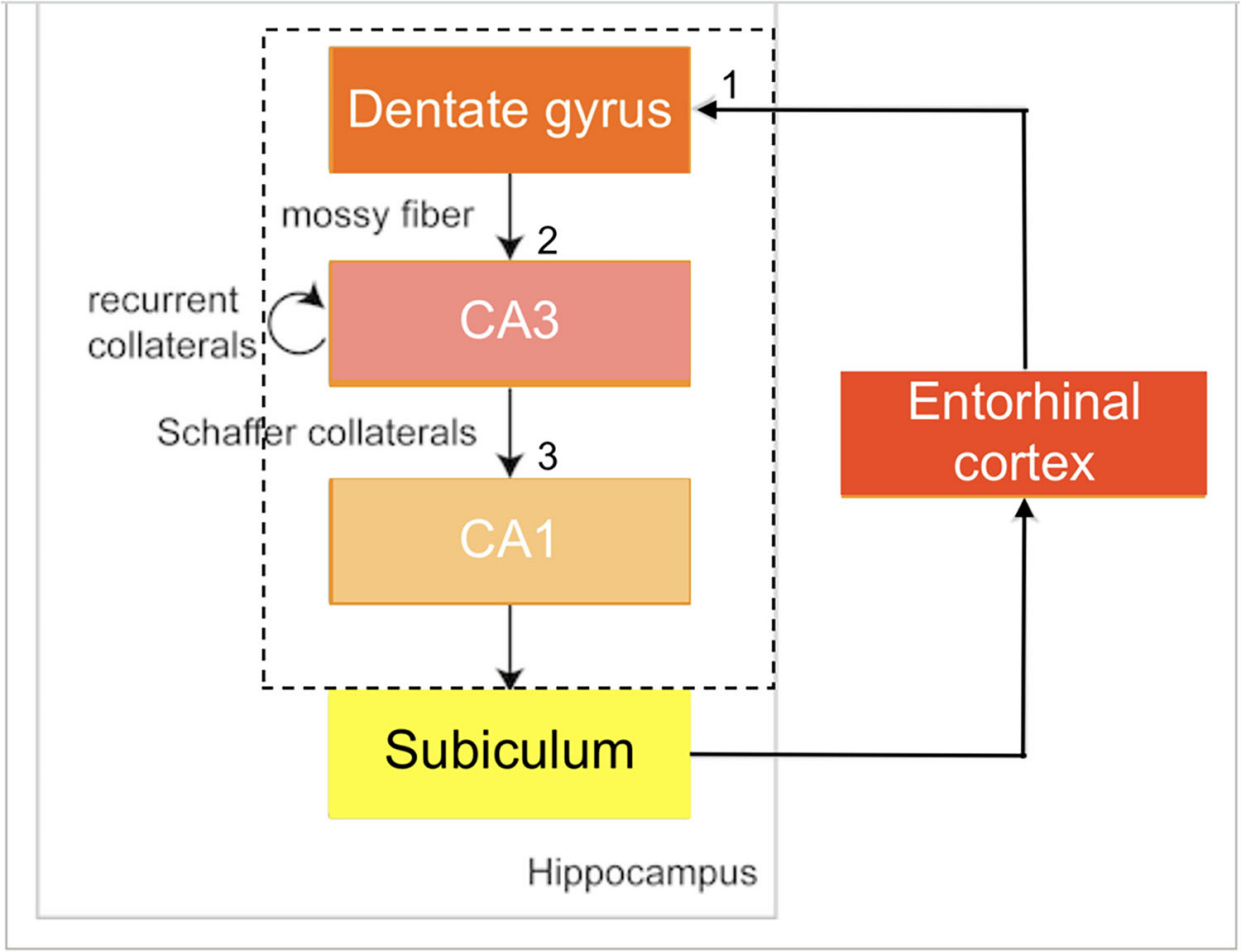
A simplified depiction of entorhinal cortex and hippocampal subfields circuitry. The trisynaptic circuit is shown with numbers 1, 2, and 3 to indicate the three synapses. The diagram omits CA2 (often included with CA3), CA4, and the continuous cortical sheet that runs from the subiculum → presubiculum → parasubiculum → medial entorhinal cortex (MEC). As part of the allocortex, the subiculum has three layers, while the PrS, PaS, and EC are in the six‐layered periallocortex (Insausti et al. 2017). Back‐projection running from the subiculum to CA1 and CA3 (Lin et al. 2021) and from CA3 to the DG (Núñez‐Ochoa et al. 2021) is not shown in the diagram. Adapted from Sun et al. (2024). Creative Commons.

The TSC (Figure 1) includes the three subregions DG, CA3, and CA1, the latter being a location for the production of theta waves, long‐term potentiation (LTP; Bliss & Lomo 1973; Bliss 2025), and long‐term depression (LTD; Mulkey and Malenka 1992; Basu and Siegelbaum 2015; Fujii et al. 2025), essential processes in the acquisition and maintenance of memories.

Palombo et al. (2018) found that the volume of a combined Region of Interest comprising the DG/CA4/CA2/3 was associated with internal details scores in autobiographic memory. Sun et al. (2025) used continuous naturalistic stimuli in the form of an audio movie, *Forrest Gump*, to study pattern separation and completion within the TSC and subiculum. The DG‐CA3 subfield pair exhibited evidence consistent with a pattern separation process, while the CA3‐CA1 and CA1‐subiculum pairs showed evidence of pattern completion (Sun et al. 2025). Sun et al. (2025) observed changes during the second half of the presentation, which indicated reduced pattern completion in the CA3‐CA1 subfield pair and increased pattern separation in the CA1‐subiculum pair. Other research on hippocampal subfields and pattern memory has focused on the presubiculum (PrS) and parasubiculum (PaS). Dalton and Maguire (2017) suggested that the PrS and PaS could function as the “hippocampal hub of the scene processing network.” They predicted that internal representations of scenes within a naturalistic 3D framework would tend to preferentially recruit the pre/parasubiculum. This prediction was confirmed by Barry et al. (2021) with a strong correlation between a combined left PrS/PaS volume and the quantity of autobiographical memory (AM) details produced over time, especially of the left side. pre/parasubiculum. A study of AM by Leelaarporn et al. (2024) also pointed to a PrS/PaS connection to episodic memory by the PrS/PaS contributing: “over and above to AM retrieval…and was strongly connected to other brain regions typically associated with AM, such as the ventromedial prefrontal cortex (vmPFC) and medial/lateral parietal regions.” The activation during AM was much stronger in the pre/parasubiculum compared with CA1, the subiculum, and DG/CA4. In an electrophysiological and molecular study, Sammons et al. (2019) suggested that the PaS contains functionally specialized cells, which produce strong theta modulation and project to layer II of the medial entorhinal cortex (MEC), suggesting a role in our ability to represent internal images of the external environment. Sammons et al. (2022) found local microcircuitry in the PaS that had both distinctive and common features of hippocampal connectivity.

To further elucidate the connections between the hippocampus and VMI, we investigated the relation between 12 hippocampal subfields (in left, right, and bilateral volumes) and the ability of healthy adults to form vivid VMI. We indexed individual differences in the ability to vividly image real‐world objects and scenes with the VVIQ (Marks 1973, 1995).

Principal component analysis (PCA) is a method for reducing large datasets into fewer variables while preserving key data trends. It simplifies data by identifying uncorrelated components that capture the most variance, making analysis faster and more efficient. PCA reduces the number of dimensions in a dataset by invoking its latent structure by transforming potentially correlated variables into a smaller set of variables, called principal components. We used PCA to investigate the relation of the trisynaptic circuit and other hippocampal zones to individual differences in VMI ability. We applied PCA to 36 hippocampal subfield volumes to determine the ability of the obtained principal components to demarcate hippocampal formation subregions that predict VMI ability indexed by vividness.

The study hypotheses were as follows:

*H1*: PCA of the 36 hippocampal subfield volumes will reveal a principal component referencing the trisynaptic circuit indexed by the DG, CA1, CA3, and CA4 subfields.
*H2*: Participants with high VVIQ scores will have larger scores for the principal component referencing the trisynaptic circuit (if one is found) than low‐VVIQ participants.
*H3*: PCA of the 36 hippocampal subfield volumes will reveal a principal component indexing the parasubiculum.
*H4*: Participants with high VVIQ scores will have larger scores for the component indexing the parasubiculum (if one is found) than low‐VVIQ participants.

## 2 Method and Materials

### 2.1 Vividness of Visual Imagery Questionnaire

All participants were tested on the VVIQ (Marks 1973, 1995). The VVIQ is a self‐report measure of the clarity and liveliness of visual imagery and aims to evoke images that vary in vividness, ambiance, and feeling. The questionnaire consists of 16 items, which participants score between 1 and 5 according to how vividly they can mentally image a family member or friend, a shop, the sky, and a countryside scene, leading to a total score between 16 and 80. The VVIQ scores were converted to percentages (VVIQ100) and squared for normalization. The instructions state the following:

“Visual imagery refers to the ability to visualize, that is, the ability to form mental pictures, or to ‘see in the mind's eye’. Marked individual differences are found in the strength and clarity of reported visual imagery and these differences are of considerable psychological interest. The aim of this test is to determine the vividness of your visual imagery. The items of the test will possibly bring certain images to your mind. You are asked to rate the vividness of each image by reference to the five‐point scale given below. For example, if your image is ‘vague and dim’, then give it a rating of 4. After each item, write the appropriate number in the box provided. The first box is for an image obtained with your eyes open and the second box is for an image obtained with your eyes closed. Before you turn to the items on the next page, familiarize yourself with the different categories on the rating scale. Throughout the test, refer to the rating scale when judging the vividness of each image. Try to do each item separately, independent of how you may have done other items. Complete all items for images obtained with the eyes open and then return to the beginning of the questionnaire and rate the image obtained for each item with your eyes closed. Try and give your ‘eyes closed’ rating independently of the ‘eyes open’ rating. The two ratings for a given item may not in all cases be the same.”

The five‐point rating scale of the VVIQ is presented in Table 1.

**TABLE 1:** The rating scale in the Vividness of Visual Imagery Questionnaire.

Rating	Description
5	Perfectly clear and as vivid as normal vision
4	Clear and reasonably vivid
3	Moderately clear and vivid
2	Vague and dim
1	No image at all, you only “know” that you are thinking of an object

The aim of the VVIQ is to assess visual imagery vividness under conditions which allow a progressive development of scenes, situations, or events as naturally as possible. The items are intended to evoke sufficient interest, meaning, and affect conducive to image generation. The 16 items are arranged in blocks of four, in which each has a theme and at least one item describing a visual image that includes motion (Table S1). Each theme provides a narrative to guide a progression of mental imagery. Participants rate the vividness of their images separately with eyes open and eyes closed.

### 2.2 MRI Analysis

The following is a condensed description of the MRI analysis described in detail elsewhere (Tabi et al. 2022). The methods, participants, data collection, and curation were contributed by Tabi et al. (2022) at the University of Oxford and are available at https://osf.io/q37vn/ accessed on June 26, 2025. Full details are available at https://www.sciencedirect.com/science/article/pii/S0010945221003488 (accessed on June 26, 2025). Younes Adam Tabi gave permission for the re‐analyses, but they did not participate in the re‐analyses or in writing this article.

A 3T Siemens Magnetom Verio syngo scanner was used to acquire T1‐weighted volumetric images through a magnetization‐prepared rapid gradient echo protocol (MPRAGE) in sagittal orientation (TR = 2000 ms, TE = 1.94 ms, TI = 880 ms, Flip angle = 8°, FOV read = 256 mm, Voxel size = 1.0 mm × 1.0 mm × 1.0 mm). The same machine was used to record T2‐FLAIR images.

FSL FIRST (Patenaude et al. 2011) was used to generate hippocampal and amygdala volume for both hemispheres from the T1 anatomical images. These volumes were corrected for age and total intracranial volume calculated using FSL SIENAX, which estimates total brain tissue volume from a single image, normalized for skull size. It strips non‐brain tissue and uses the brain and skull images to estimate the scaling between the subject's image and standard space. It then runs tissue segmentation to estimate the volume of brain tissue, and multiplies this by the estimated scaling factor, to reduce head size‐related variability between subjects. Hippocampal subfields were calculated through the standard FreeSurfer pipeline (cortical reconstruction and volumetric segmentation: http://surfer.nmr.mgh.harvard.edu/  (accessed on October 18, 2025) corrected for age and total intracranial volume based on T1 and T2 image inputs.

### 2.3 Availability of Data

All of the original raw data are provided at https://osf.io/q37vn/ (accessed on October 18, 2025).

### 2.4 Participants

Participants gave their informed consent to be involved in the study, which was approved by the local ethics committee at the University of Oxford, United Kingdom (Tabi et al. 2022). The sample consisted of 53 healthy, middle‐ to older‐aged participants who completed an MRI scan, a short‐term memory task (not included here), the VVIQ, and the Addenbrooke's Cognitive Examination, third edition (ACE‐III), a widely used structured cognitive assessment (So et al. 2018). Following the extreme groups protocol, it was decided in advance to select and compare subgroups consisting of the 10 highest VVIQ and 10 lowest VVIQ scorers. A gap of 21 points separated the low VVIQ (16–52) and the high VVIQ (73–80) groups. The high VVIQ group contained six females. The low VVIQ group contained five females, one having a minimum score of 16 (a so‐called “aphantasic” who rated all 16 VVIQ items at level one—“No image at all, you only ‘know’ that you are thinking of an object”).  Table 2 describes the samples of participants who were predominantly right‐handed and reported no history of neurological or psychiatric disorders. Demographic information, including age and gender, was collected through self‐report questionnaires administered prior to testing. The current analysis utilized 100% of the available dataset without any data or subjects deleted. While the data may have contained outliers as a consequence, the policy of non‐deletion avoided the risk of bias due to data selection.

**TABLE 2:** The sample sizes, gender split, age means and SD, and VVIQ mean scores and SD.

Sample	*N*	Female:Male	Age mean (SD)	VVIQ mean (SD)
Whole sample	53	29:24	68.00 (7.20)	62.23 (12.53)
High VVIQ group	10	6:4	67.40 (7.83)	72.90 (9.46)
Low VVIQ group	10	5:5	68.70 (6.77)	44.50 (15.51)

### 2.5 Principal Components Analysis

The SPSS (version 31.0.1.0 49) dimension reduction procedure for PCA was applied to the complete set of 36 subfield volumes (12 left, 12 right, and 12 bilateral volumes). To avoid subjectivity in selecting the number of components to retain, we adopted the Kaiser–Guttman criterion. For *N* of 53, the Kaiser–Guttman criterion would be expected to minimize any risk of overestimating the number of components, which may occur with *N* < 30 (Weeraratne et al. 2025). The program extracted all factors with eigenvalues greater than 1.0 (the Kaiser–Guttman criterion) and performed a Varimax Rotation (delta = 0, Kappa = 4) with Kaiser normalization and a maximum of 25 iterations, with seven iterations finally required. Coefficients were sorted by size, and coefficients below 0.10 were suppressed. Factor scores were saved as regression variables for further analysis.

## 3 Results

### 3.1 Data Quality Control

FreeSurfer hippocampal subfield volumes depend on the reliability and validity of the correction system used to calibrate measures against head size differences (Perlaki et al. 2014; Pintos‐Allo et al. 2025). To check the reliability of the obtained subfield volume scores, the mean volumes from the 53 subjects were compared to the values published in a reference study at the Max Planck Institute of Psychiatry (MPIP) with 614 subjects (Sämann et al. 2022). The values in the current dataset were highly similar to those in the reference set, with only one minor discrepancy for the molecular layer subfield volume, which was approximately one standard deviation lower than the reference value (Figure S1). The Spearman's rank correlation coefficient between the mean subfield volume ranks for the study sample and the reference sample was 0.986 with *t*(10) = 18.71, *p* < 0.000001.

### 3.2 Principal Components Analysis

Seven components with an eigenvalue > 1.0 accounted for 84.64% of the variance (Table 3). The analysis was completed in eight rotations. The analysis was repeated without the bilateral subfield volumes, yielding the same principal components. Tables S2–S4 present descriptive statistics, total variances explained, and the rotated component matrix with scores above 0.25.

**TABLE 3:** Varimax‐rotated component matrix showing loadings above 0.1.

Subfield	C 1	C 2	C 3	C 4	C 5	C 6	C 7
bilateral_CA4	0.916	0.191	0.106	0.105		0.170	
right_CA4	0.903	0.106		0.157			0.154
bilateral_CA3	0.885	0.136	−0.121	−0.109		−0.136	−0.230
bilateral_GC‐ML‐DG	0.853	0.236		0.304		0.164	
right_GC‐ML‐DG	0.835	0.150		0.308			
right_CA3	0.814		−0.201			−0.250	−0.170
left_CA4	0.808	0.246	0.139			0.276	
left_CA3	0.771	0.274		−0.171	0.177		−0.245
left_GC‐ML‐DG	0.771	0.290	0.132	0.266		0.285	
bilateral_CA1	0.689	0.142	−0.122	0.348	0.197	0.470	
right_CA1	0.652		−0.182	0.317	0.225	0.369	0.161
left_CA1	0.638	0.182		0.333	0.148	0.501	
bilateral_molecular_layer	0.177	0.821	−0.181	0.159	0.290		
left_molecular_layer	0.199	0.820	−0.138	0.144	0.213	0.111	−0.123
right_hippocampal_tail	0.357	0.815		−0.105		−0.178	
bilateral_presubiculum		0.811	0.417			0.255	
right_presubiculum		0.788	0.402			0.211	
bilateral_hippocampal_tail	0.452	0.772	0.149	−0.148	0.104	−0.161	
left_presubiculum		0.759	0.393			0.279	
right_molecular_layer	0.137	0.726	−0.200	0.154	0.326		
right_subiculum	0.186	0.704	0.224	0.143		0.502	0.162
bilateral_subiculum	0.243	0.635	0.210	0.213		0.629	
left_hippocampal_tail	0.500	0.625	0.188	−0.177	0.160	−0.121	
bilateral_parasubiculum		0.133	0.950				
right_parasubiculum		0.183	0.825				0.124
left_parasubiculum			0.811				
bilateral_fimbria	0.222		0.100	0.907	−0.228	0.113	
left_fimbria	0.199			0.824	−0.337	0.118	0.106
right_fimbria	0.205	0.235	0.204	0.822			
bilateral_hippocampal‐fissure		0.153		−0.165	0.949		
left_hippocampal‐fissure		0.151	−0.119		0.910		0.125
right_hippocampal‐fissure	0.173	0.134		−0.219	0.861		
left_subiculum	0.269	0.461	0.162	0.258		0.675	
bilateral_HATA							0.984
right_HATA	−0.111	0.111				−0.201	0.849
left_HATA				−0.125		0.404	0.642

### 3.3 Description of Components

**FIGURE 2:**
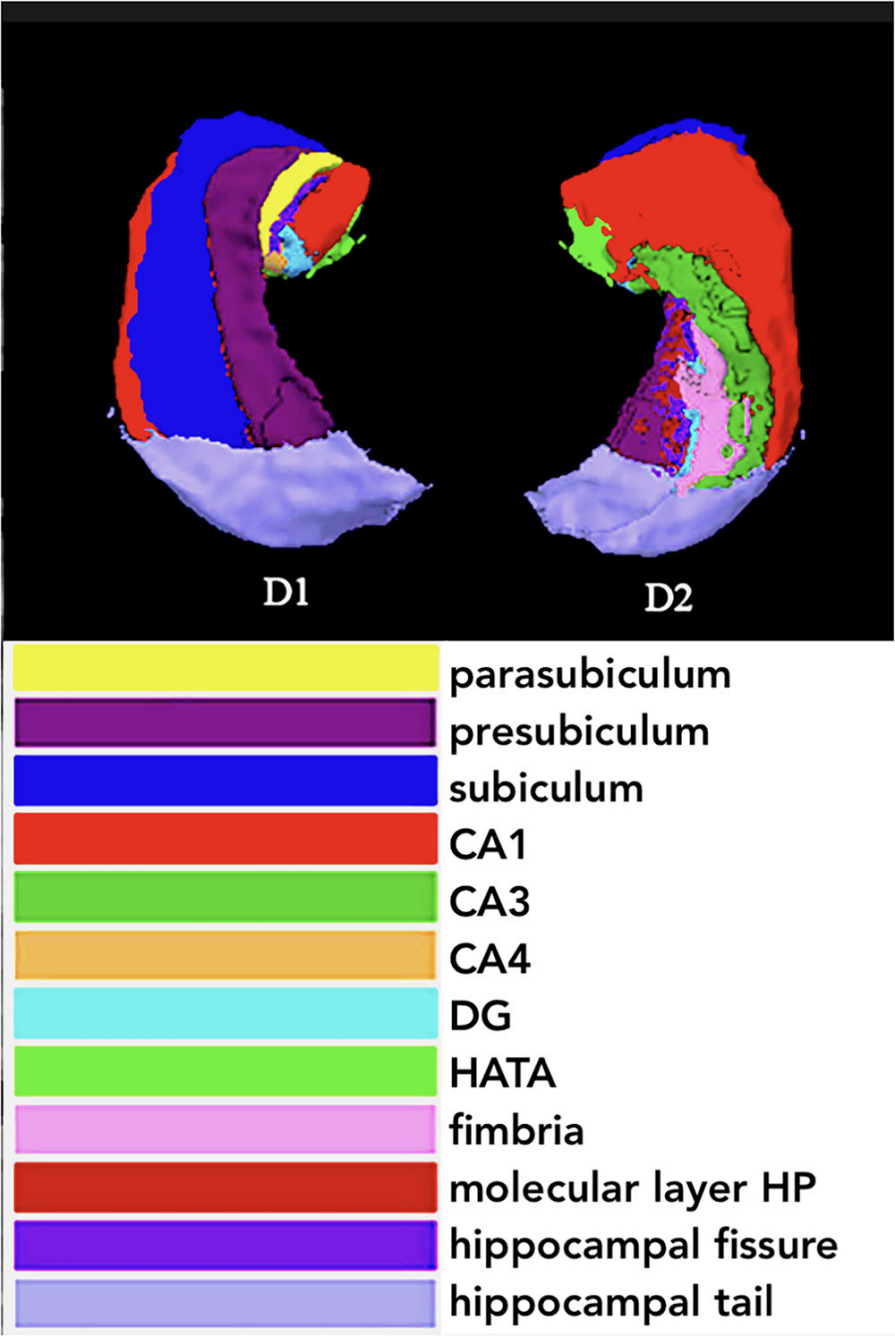
The hippocampal subfields analyzed in the PCA. Illustration adapted from He et al. (2022). Creative Commons.

The seven components are described in Table 4. The morphometry of the 12 constituent volumes is shown in Figure 2. For each component, the volume of the constituent subfields was computed as a proportion of the entire hippocampal volume (Table 4).

**TABLE 4:** The seven principal components, variance explained, suggested name, referenced subfield volumes, referenced volumes as a proportion of the total hippocampal volume (HV), standard deviation of referenced proportion, and brief description.

Component and variance explained	Name	Referenced subfield volumes	Referenced volume proportion (SD)	Brief description
C 1. 36.867	Trisynaptic circuit	Granule cell layer/molecular layer/dentate gyrus (GC‐ML‐DG), CA1, CA3, and CA4	0.413 (0.021)	Internal gray matter loop responsible for pattern separation (DG), pattern completion (CA3), and final processing for detail (CA1).
C 2. 14.254	Periallocortical interface	Molecular layer, hippocampal tail, presubiculum, and subiculum	0.491 (0.021)	Function is uncertain. A transitional, output zone between the hippocampal formation and the surrounding cortex.
C 3. 12.044	Parasubiculum	Parasubiculum and presubiculum	0.018 (0.003)	Inputs from subiculum, neocortex and anterior thalamus. Projects to entorhinal cortex.
C 4. 7.152	Fimbria	Fimbria	0.017 (0.006)	Gateway for subcortical and cortical projection via the fornix.
C 5. 6.412	Hippocampal fissure (sulcus)	Hippocampal fissure (sulcus)	0.045 (0.007)	A boundary separating the DG from the subiculum and CA fields.
C 6. 4.691	Subiculum	Subiculum and CA1	0.122 (0.007)	Projects to cortical and subcortical targets.
C 7. 3.219	Hippocampal amygdaloid transition area (HATA)	HATA	0.017 (0.003)	Serves as the transition area between the hippocampus and the amygdala.

**FIGURE 3:**
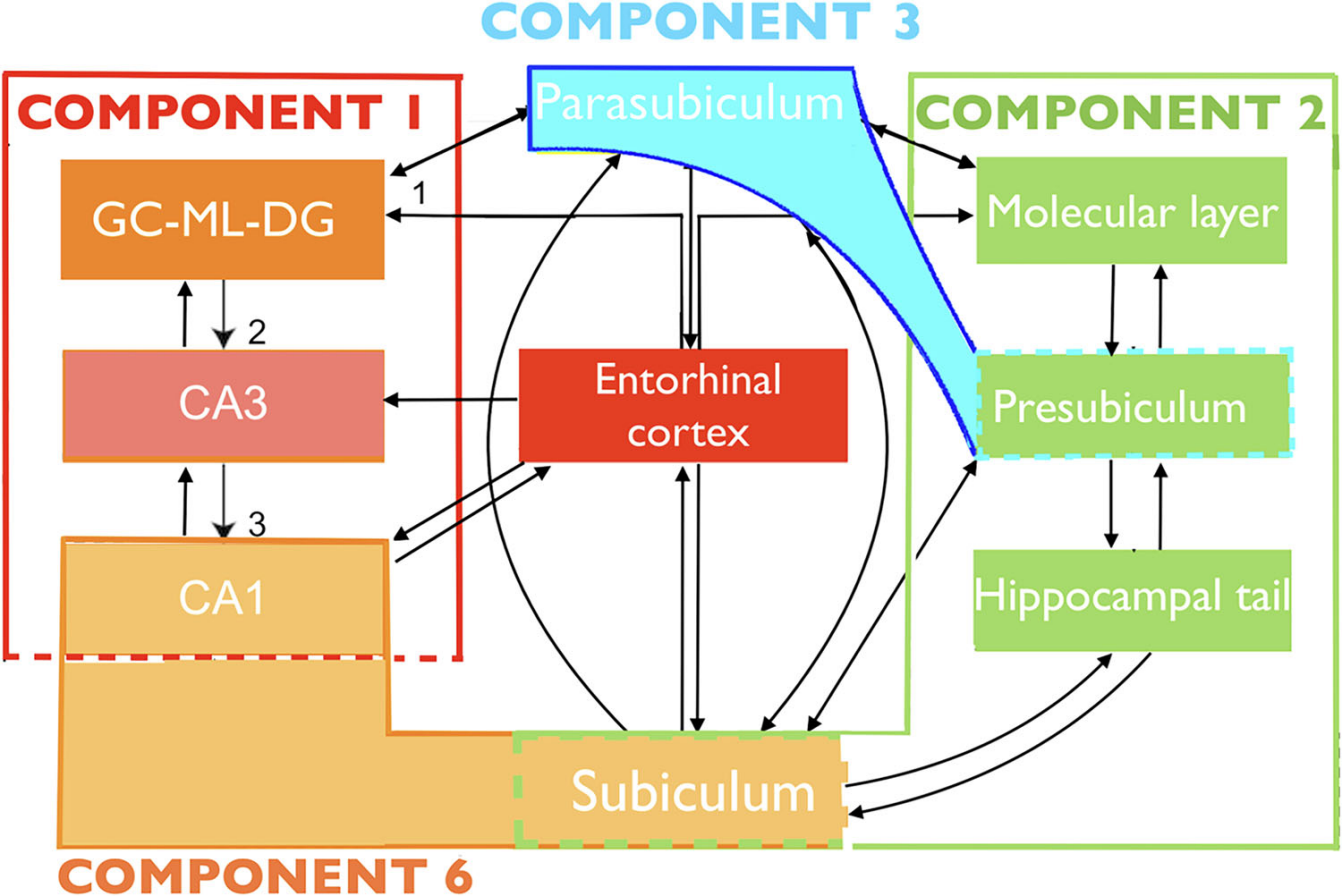
Schematic depiction of a PCA representation of a pathway in the hippocampal formation beginning with the trisynaptic circuit (C 1), then a subregion that includes both CA1 and the subiculum, which is cross‐loaded on C 2 and C 6, continues to a subregion that references the subiculum, presubiculum, molecular layer and the hippocampal tail (C 2) and finally to the parasubiculum (C 3, receiving a cross‐loading from the presubiculum). Arrows, indicating neural connections, are used sparingly, based on Andersen (2007, fig. 3.1).

Cs 1 and 2 jointly explained 51.1% of the total subfield volume variance. In this sample, the eight subfields referenced by these two components occupied 90.4% of the mean hippocampal volume. In many cases, subfields were strongly loaded (> 0.500) on a single rotated component. In three cases, cross‐loading occurred. The subiculum cross‐loaded onto C 2 (loading = 0.635) and C 6 (loading = 0.629), CA1 cross‐loaded onto C 1 (loading = 0.689) and C 6 (left CA1 loading = 0.501), and the presubiculum cross‐loaded on Cs 2 (loading = 0.811) and 3 (loading = 0.417). The cross‐loadings created a pathway linking Cs 1, 2, 6, and 3 (Figure 3).

### 3.4 Correlations Between Principal Component and VVIQ Scores

Table 5 presents the correlations between the seven component scores and VVIQ scores for the 53 study participants. Uniquely, the values for C 1 correlated at a statistically significant level with the participants’ VVIQ scores (*r* = 0.47, *r*‐square = 0.22, *p* < 0.001). For all other components, the correlations were low or close to 0.

**TABLE 5:** Pearson correlation coefficients between VVIQ scores and the principal component scores for C 1–C 7.

		C 1	C 2	C 3	C 4	C 5	C 6	C 7
	Coefficient	0.471	0.020	0.217	0.156	0.093	0.078	0.020
	*p* level (2‐tailed)	< 0.001	0.888	0.119	0.265	0.506	0.579	0.888

### 3.5 ANOVA of High and Low VVIQ Groups’ Component Scores

**FIGURE 4:**
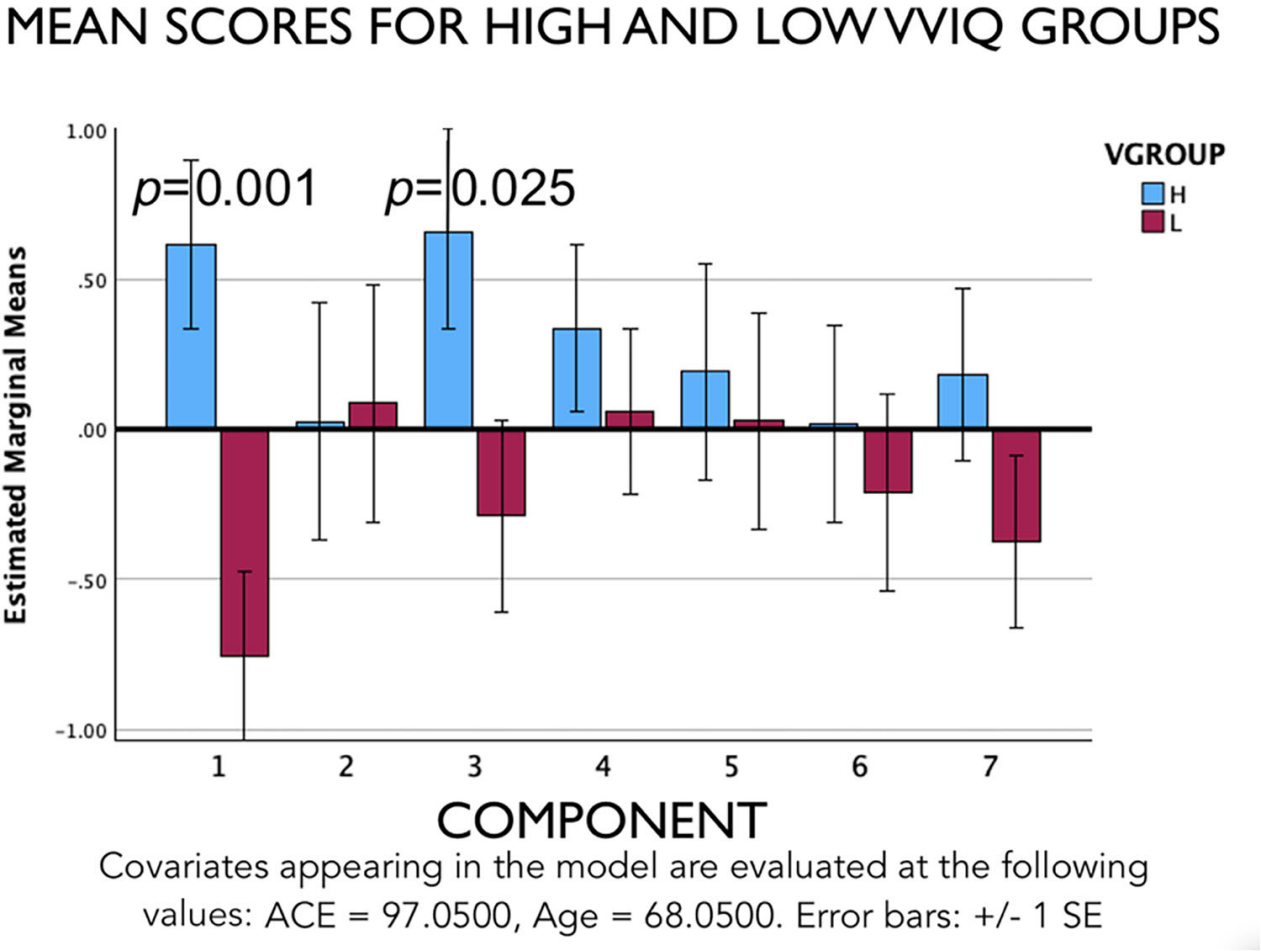
Mean scores for each component and VVIQ group. *p* values are for post hoc *t*‐tests.

In a mixed model ANOVA, we compared the group of 10 subjects with the highest VVIQ scores to the group of 10 subjects with the lowest VVIQ scores, with the seven components as a within‐subjects factor, VVIQ group as a between‐subjects factor, and ACE‐III score and Age as covariates. The component factor and its interaction with VVIQ group were nonsignificant effects. The VVIQ group factor was significant, but the ACE‐III group factor missed significance. The ANOVA within‐ and between‐subjects effects are summarized in Tables S5 and S6. Figure 4 shows the mean component scores for the high and low VVIQ groups.

### 3.6 Component Scores for Subjects at the Tail Ends of the VVIQ Distribution

**FIGURE 5:**
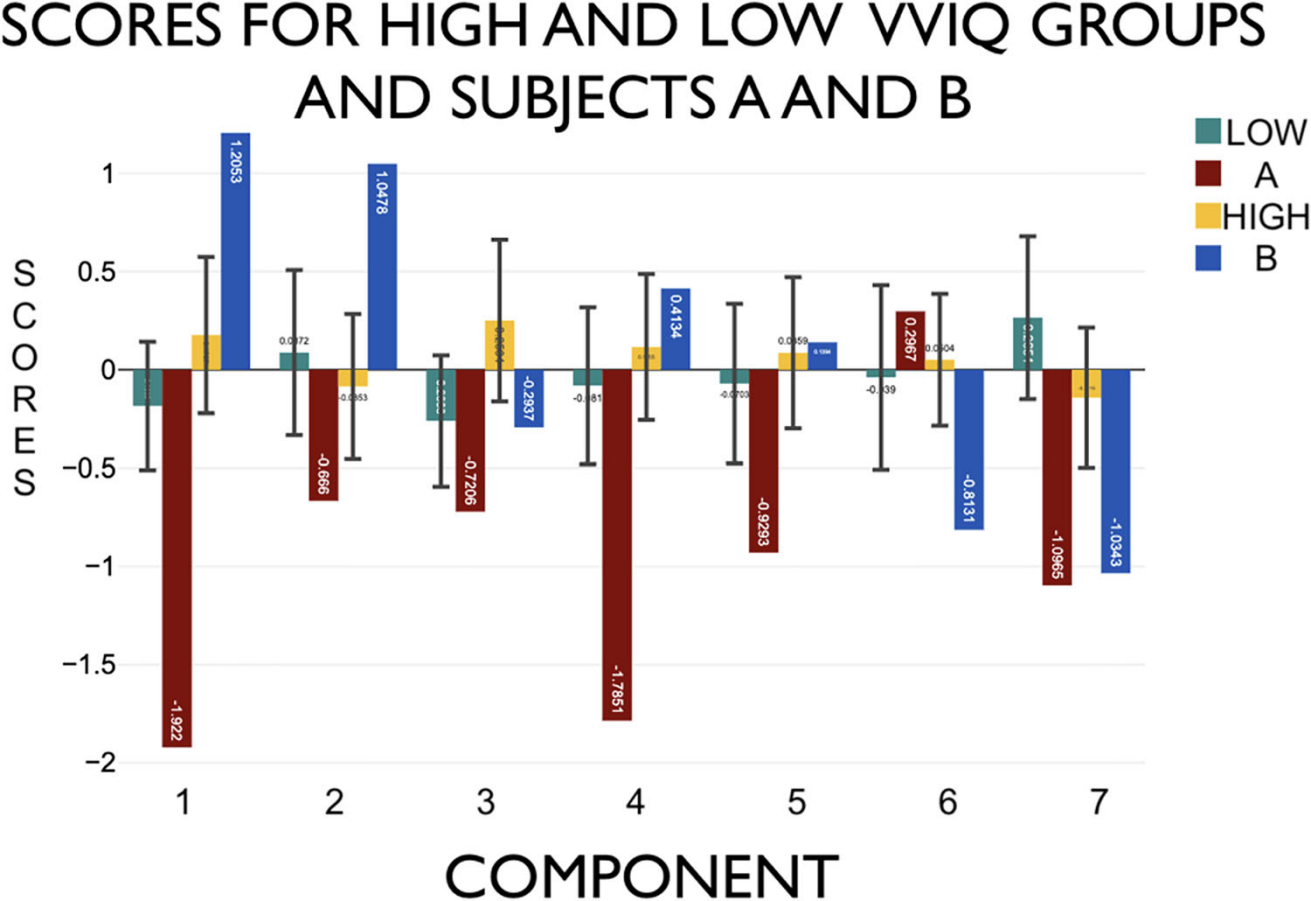
The component scores for the “aphantasic” (Subject A), “hyperphantasic” (Subject B), and the low and high VVIQ group means. Bars represent 95% confidence intervals.

The sample contained a so‐called “aphantasic” subject with a minimum VVIQ100 score and a “hyperphantasic” subject with a maximum VVIQ100 score. Figure 5 shows the subfield volume profile of the two subjects in comparison to the high and low VVIQ group mean scores.

For all components except C 6, A's scores were below the 95% confidence interval for the low VVIQ group (Figure 5). As expected, Subject B's scores were above the high VVIQ group's 95% confidence intervals for C 1 and C 2, but below the confidence intervals for C 3, C 6, and C 7. The small and negative C 3 loading for Subject B was inconsistent with H 4.

More evidence on the exceptionality of Subject A was obtained by computing prediction intervals using linear regression of VVIQ scores against each component score (Table 6). For every component, the lower bound of the predictive interval lay above zero, providing strong statistical evidence that A's score was significantly lower than the expected distribution for the low VVIQ scoring population (at either *p* < 0.01 or < 0.001 levels).

**TABLE 6:** The lower bound, LICI, and upper bound, UICI, of the prediction interval for each component for Subject A. The *p* value is for the difference between the subject's score and the predictive interval at each component.

C	LICI	UICI	*p*
1	5.84	103.17	< 0.01
2	2.07	141.88	< 0.001
3	6.17	145.53	< 0.01
4	12.52	121.05	< 0.01
5	0.65	140.42	< 0.001
6	2.07	141.88	< 0.001
7	1.41	142.21	< 0.001

The results for Subject B were less striking and nonsignificant. Subject B's score was only a short distance from the high VVIQ group mean and did not exceed the prediction interval. However, a paired *t*‐test between the component scores for Subjects A and B was statistically significant on a one‐tail test: *t*(6) = 1.96, *p* = 0.049.

## 4 Discussion

### 4.1 The Components

This study utilized PCA to investigate the volumetric features of an intensively researched brain region, the hippocampal formation. The PCA defined the structure of the canonical TSC in C 1, a previously unidentified, “quiet” region in C 2, and five other components. C 1 related to 12 subfields belonging to the TSC, namely the bilateral, left and right volumes comprising subfields GC‐ML‐DG, CA1, CA2 and CA3. As hypothesized by H1, all 12 designated TSC subfield volumes were highly loaded on C 1. Supporting H2, the high VVIQ group was found to yield significantly larger scores on C 1 than the low VVIQ group. Supporting H3, C 3 received high loadings from the PaS. Supporting H4, the high VVIQ group yielded a significantly larger mean score on C 3 than the low VVIQ group.

In consistency with H2, the strong association between participants’ VVIQ scores and their TSC sub‐volumes suggests that larger TSC volume supports more vivid conscious VMI experience (subject to the usual proviso about correlation and causation). This result confirms previously reported associations between VVIQ scores and the volumes for CA1, CA3, CA4 and GC‐ML‐DG as way stations of the TSC (Tabi et al. 2022; Marks 2025). In addition to voluntary VMI, and pending further investigation, C 1 subfields likely support unconscious mental imagery (Nanay 2023) and the dream imagery of sleep (Llewellyn 2013). A large body of evidence to date suggests that it would be impossible to construct and utilize scenes in episodic, autobiographical memory and future mental imagery without the TSC as a universal system for mental imagery production.

Of some potential significance was the finding of C 2, which references a newly identified region of four neighboring subfields: the molecular layer, hippocampal tail, PrS, and subiculum. All four subfields had strong loadings between 0.625 and 0.821 on C 2, which pinpointed a cohesive structure performing as an integrated network. Together, these four subfields occupied 49% of the total hippocampal volume for the subjects in the study sample. The molecular layer, subiculum, and presubiculum maintain their characteristic structure throughout the entire length of the hippocampal formation. There is anatomical contiguity of the four subregions in a continuum running longitudinally from the body to the tail. The functions served by C 2 remain uncertain, although multiple possibilities exist that would not entail conscious VMI, which was dissociated from C 2. C 2 references a zone where input via the molecular layer and presubiculum is received from areas that support both egocentric and allocentric coding (Wyss and Van Groen 1992; Amaral et al. 2007; Witter 2007; Agster and Burwell 2013; Rolls 2025). Output via the subiculum and hippocampal tail could be relayed as integrated, allocentric representations across the hippocampal dorsal commissure (Gloor et al. 1993), entorhinal cortex, and cerebral cortex more widely (Amaral and Witter 1989; O'Mara et al. 2001). Detailed investigation of the organization, connectivity, and function of the region referenced by C 2 is necessary before any definite conclusions are possible.

C 3 explained 12% of the variance in referencing the PaS sub‐volume with a smaller loading from PrS. The high and low VVIQ groups yielded different mean scores for C 3 at a statistically significant level (*p* = 0.025). Although weaker than the effect obtained for C 2, the finding is consistent with those of Dalton and Maguire (2017), Barry et al. (2021), and Leelaarporn et al. (2024).

C 4 explained 7% of the variance in referencing the fimbria. C 4 is another component where the high VVIQ group yielded larger component scores than the low VVIQ group but, on this occasion, to a statistically nonsignificant level. In connecting the hippocampus to the rest of the Papez circuit, the fimbria are crucial to the consolidation of short‐term to long‐term memory, as well as spatial memory and navigation (Dahmani et al. 2018, 2020).

C 5 referenced the hippocampal fissure in explaining 6% of volume variance. The hippocampal fissure is a sulcus, which does not store memories or support cognition. The slight, nonsignificant, mean volume difference between the low and high VVIQ groups for this component can be attributed to chance.

C 6 received high loadings from the subiculum and CA1 volumes, explaining 5% of subfield volume space. As noted in the Introduction, the CA1‐subiculum subfield pair has evidenced pattern completion (Sun et al. 2025), while the subiculum has been implicated in separating similar memories (Molitor et al. 2021) and detail production (Palombo et al. 2018). CA1 has repeatedly been implicated as a center for the production of theta waves, LTP, and LTD for the acquisition and maintenance of memories (see Section 1).

C 7 referenced the transition area between the hippocampus and the amygdala, HATA, explaining 3.2% of variance. The affective elements to scene construction can evoke the complete vista of emotional responses, necessarily involving the Papez circuit and the direct hippocampal‐amygdaloid connections via HATA, as acknowledged in the author's VMI theory (Marks 2023b; see further details below).

### 4.2 Subjects A and B

The component scores of Subject A were notably different from their peers with scores for Cs 1, 3, 4, and 5 that were considerably more extreme. For Cs 2 and 7, A's scores again diverged from their peer group but on the opposite side of the abscissa to the low VVIQ group. The low VVIQ group mean of 44.50 (SD = 15.51) was toward the middle of the VVIQ scale while the high VVIQ group mean of 72.90 (SD = 9.46) was close to the maximum VVIQ score (80) so that Subject B's score was just seven points higher than the high VVIQ group mean. B's scores lay above the 95% confidence interval for C 1 and C 2 but below these for C 3, C 6, and C 7. For C 7, referencing the volumes for the HATA, A and B both scored close to −1.00. These findings need to be followed up with larger samples of extreme‐scoring subjects.

### 4.3 Connections With Recent Theorizing

Consistent with the recent empirical and theoretical literature, the present findings indicate that the TSC and PaS systems both have a strong connection with VMI vividness. This finding is supported by the view of Hwang and Lee (2024) that the hippocampus concurrently engages in scene construction (binding elements of “what” and “where”) and the temporal organization of memory (“when”). Hwang and Lee suggest that neural markers of scene construction are spontaneously induced and functionally linked to successful temporal order memory retrieval as the hippocampus detects boundaries separating one context from another using shifting representational scales along its longitudinal axis (Maurer and Nadel 2021). A theory by Bakermans et al. (2025) suggests that the hippocampus supports constructive reasoning by building compositional memories. Accordingly, hippocampal responses include a process of binding existing building blocks—or “primitives”—of experience. This ability to compose and to contextualize episodes and events enables inferences about the layout of new environments without requiring any new learning. The VVIQ items utilized in the present study consist of multiple unfolding of actions within scenes, which exemplify the agile, flexible, and rapid scene construction that the TSC appears designed to achieve. In similar fashion, Rolls’ (2025) network model attempts to explain how scene representations are built in the ventromedial visual pathway to the hippocampus. Scene representations are formed by “stitching together” visual fixation scene patches using a continuous “attractor network” in the hippocampus and para‐hippocampal cortex. Each neuron is assumed to act as a spatial view cell, responding to a location in a viewed scene based on visual features. Rolls’ theory emphasizes the anchoring of hippocampal scene representations in allocentric space, or points of view, which appear crucial for navigation and episodic memory in any new or previously visited location.

The author proposes a system of six modules for visual perception and VMI comprising *Objects*, *Schemata*, *Actions*, *Affect*, *Goals* and *Other's Actions* (Marks 2023b). Serving each of these six modules, and working closely with the cerebellum, the hippocampus plays an essential coordinating role. Schemata are proposed as key processes to the structure and smooth flow of actions by serving as mental models or templates for rapid processing within allocentric perceptual‐motor systems including our ability to navigate the environment while maintaining balance, posture, and a steadfast gait. Unconscious schemata are constructed and coordinated across the hippocampal region in conjunction with several cortical regions including the precuneus (Duan et al. 2025) and other scene‐selective regions such as the occipital place area (OPA), the retrosplenial complex (RSC), and the parahippocampal place area (PPA) (Tullo et al. 2022, 2023) divided between posterior and anterior networks (Watson and Andrews 2024) and subcortical regions, especially the cerebellum (Zeidler et al. 2020; Rondi‐Reig et al. 2022). This modular approach is supported by findings on the interactions between the PPA, RSC, and OPA using functional magnetic resonance data (Tullo et al. 2022). Tullo et al. tested whether resting‐state connectivity among scene‐selective regions reflected individual differences in VMI vividness using the VVIQ. An inhibitory influence of the occipito‐medial region on temporal regions was observed, as well as an excitatory influence of more anterior, medial, and posterior brain regions. The connection strength from OPA to PPA was especially high in the left hemisphere, with the signal between these regions showing positive correlations with vivid VMI ability.

Pending further investigation, it is speculated that the subregion referenced by C 2 may serve the creation, coordination, and control of schemata, which are hypothesized to underlie action, speech, and performance (Marks 2023b).

## 5 Conclusions

1. In a dataset with 36 hippocampal subfield volumes from 53 healthy adults, a PCA identified seven principal components.
2. A strong association was found between scores on Component 1, referencing the trisynaptic circuit, and the vividness of conscious VMI, indexed by the VVIQ.
3. A weak association was found between scores on Component 3, referencing the parasubiculum, and conscious VMI vividness, indexed by the VVIQ.
4. Component 2 referenced a large, previously unidentified, subregion consisting of the molecular layer, subiculum, presubiculum, and hippocampal tail. This subregion comprised 49% of the hippocampal formation volume and had no association with the vividness of conscious VMI, indexed by the VVIQ.
5. The subregion referenced by Component 2 may serve a “backroom” role, creating, integrating, and controlling nonconscious “action maps” or schemata necessary for keeping track of contextual equivalence and nonequivalence decision‐making—vital for action, speech, and overt performance, as well as episodic memory. Only further investigation will tell.

## Author Contributions

**David F. Marks**: conceptualization, writing – original draft, methodology, validation, visualization, writing – review & editing, software, formal analysis, project administration, supervision.

## Funding

The author has nothing to report.

## Supporting information




**FIGURE S1**. The mean subfield volumes obtained in the current study (red circles) together with subfield volumes from a reference study at the Max Planck Institute of Psychiatry (MPIP) with *N*  =  614 subjects (T1WI, FS6.0, mean values and 1 *SD*) (black squares) ranked according to their average size. The authors claim the volume ranking order is extremely robust across other samples including 3 Tesla samples (not shown). The colored frames point to subregions that underlie ranking violation rules. With the exception of the value for the molecular layer, which appears on the borderline of 1 SD, the current study's values and rankings fall inside the 1 SD band of each reference mean volume. The Spearman correlation coefficient, rho, between the sample and reference points is 0.986, p < 0.001. Adapted from Sämann et al. (2022). Creative Commons.

TABLE S1. Items in the Vividness of Visual Imagery Questionnaire (Marks 1973, 1995).

TABLE S2. PCA Statistics Tables.

TABLE S3. PCA Statistics Tables.

TABLE S4. PCA Statistics Tables.

TABLE S5. Mixed model ANOVA summary tables with Component scores as the dependent variable Within‐subjects effects summary table.

TABLE S6. Mixed model ANOVA summary tables with Component scores as the dependent variable Between subjects effects summary table.
