# Supplementary material for: Principal Components Analysis Relates Hippocampal Trisynaptic Circuit to Vividness of Visual Mental Imagery
Source: Brain Behav. 2026 Aug 30;16(9):e71666. doi: 10.1002/brb3.71666 (PMC13526228; doi:10.1002/brb3.71666)
Supplement: Supplementary file 1 [file BRB3-16-e71666-s002.docx]

**TABLE S5**

**Mixed model ANOVA summary tables with Component scores as the dependent variable**

**Within-subjects effects summary table**

| Source | | Type III Sum of Squares | df | Mean Square | F | Sig. | Partial Eta Squared | Noncent Parameter | Observed Power |
| --- | --- | --- | --- | --- | --- | --- | --- | --- | --- |
| Component | Sphericity Assumed | 4.882 | 6 | .814 | .773 | .593 | .046 | 4.638 | .293 |
|  | Greenhouse-Geisser | 4.882 | 4.86 | 1.005 | .773 | .569 | .046 | 3.756 | .260 |
|  | Huynh-Feldt | 4.882 | 6.00 | .814 | .773 | .593 | .046 | 4.638 | .293 |
|  | Lower-bound | 4.882 | 1.00 | 4.882 | .773 | .392 | .046 | .773 | .131 |
| Component  by Age | Sphericity Assumed | 1.574 | 6 | .262 | .249 | .959 | .015 | 1.495 | .114 |
|  | Greenhouse-Geisser | 1.574 | 4.86 | .324 | .249 | .936 | .015 | 1.211 | .107 |
|  | Huynh-Feldt | 1.574 | 6.00 | .262 | .249 | .959 | .015 | 1.495 | .114 |
|  | Lower-bound | 1.574 | 1.00 | 1.574 | .249 | .624 | .015 | .249 | .076 |
| Component  By -III | Sphericity Assumed | 6.200 | 6 | 1.033 | .982 | .442 | .058 | 5.890 | .371 |
|  | Greenhouse-Geisser | 6.200 | 4.86 | 1.276 | .982 | .433 | .058 | 4.769 | .328 |
|  | Huynh-Feldt | 6.200 | 6.00 | 1.033 | .982 | .442 | .058 | 5.890 | .371 |
|  | Lower-bound | 6.200 | 1.00 | 6.200 | .982 | .337 | .058 | .982 | .154 |
| Component  by VVIQ Group | Sphericity Assumed | 7.181 |  | 1.197 | 1.14 | .347 | .066 | 6.822 | .429 |
|  | Greenhouse-Geisser | 7.181 | 4.86 | 1.478 | 1.14 | .348 | .066 | 5.525 | .378 |
|  | Huynh-Feldt | 7.181 | 6.00 | 1.197 | 1.14 | .347 | .066 | 6.822 | .429 |
|  | Lower-bound | 7.181 | 1.00 | 7.181 | 1.14 | .302 | .066 | 1.137 | .171 |
| Error  (Component) | Sphericity Assumed | 101.05 | 96 | 1.053 |  |  |  |  |  |
|  | Greenhouse-Geisser | 101.05 | 77.7 | 1.300 |  |  |  |  |  |
|  | Huynh-Feldt | 101.05 | 96.0 | 1.053 |  |  |  |  |  |
|  | Lower-bound | 101.05 | 16.0 | 6.316 |  |  |  |  |  |
