# Supplementary material for: Principal Components Analysis Relates Hippocampal Trisynaptic Circuit to Vividness of Visual Mental Imagery
Source: Brain Behav. 2026 Aug 30;16(9):e71666. doi: 10.1002/brb3.71666 (PMC13526228; doi:10.1002/brb3.71666)
Supplement: Supplementary file 2 [file BRB3-16-e71666-s003.docx]

**TABLE S3**

**PCA Statistics Tables**

| **Total Variance Explained** | | | | | | | | | | | |
| --- | --- | --- | --- | --- | --- | --- | --- | --- | --- | --- | --- |
| Component | Initial Eigenvalues | | | | Extraction Sums of Squared Loadings | | | Rotation Sums of Squared Loadings | | | |
|  | Total | % of Variance | | Cumulative % | Total | % of Variance | Cumulative % | Total | % of Variance | Cumulative % |  |
| 1 | 13.272 | 36.867 | 36.867 | | 13.272 | 36.867 | 36.867 | 8.707 | 24.187 | 24.187 |  |
| 2 | 5.131 | 14.254 | 51.121 | | 5.131 | 14.254 | 51.121 | 7.115 | 19.763 | 43.950 |  |
| 3 | 4.336 | 12.044 | 63.165 | | 4.336 | 12.044 | 63.165 | 3.267 | 9.075 | 53.025 |  |
| 4 | 2.575 | 7.152 | 70.316 | | 2.575 | 7.152 | 70.316 | 3.263 | 9.065 | 62.090 |  |
| 5 | 2.308 | 6.412 | 76.729 | | 2.308 | 6.412 | 76.729 | 3.133 | 8.704 | 70.794 |  |
| 6 | 1.689 | 4.691 | 81.420 | | 1.689 | 4.691 | 81.420 | 2.539 | 7.052 | 77.845 |  |
| 7 | 1.159 | 3.219 | 84.639 | | 1.159 | 3.219 | 84.639 | 2.446 | 6.794 | 84.639 |  |
