# Supplementary material for: Principal Components Analysis Relates Hippocampal Trisynaptic Circuit to Vividness of Visual Mental Imagery
Source: Brain Behav. 2026 Aug 30;16(9):e71666. doi: 10.1002/brb3.71666 (PMC13526228; doi:10.1002/brb3.71666)
Supplement: Supplementary file 3 [file BRB3-16-e71666-s004.docx]

**TABLE S1**

**Items in the Vividness of Visual Imagery Questionnaire (Marks, 1973, 1995)**

For items 1 to 4, think of some relative or friend whom you frequently see (but who is not with you at present) and consider carefully the picture that comes before your mind’s eye.

1 The exact contour of face, head, shoulders, and body.

2 Characteristic poses of head, attitudes of body, etc.

3 The precise carriage, length of step, etc. in walking.

4 The different colors worn in some familiar clothes.

Visualize a rising sun. Consider carefully the picture that comes before your mind’s eye.

5 The sun is rising above the horizon into a hazy sky.

6 The sky clears and surrounds the sun with blueness.

7 Clouds. A storm blows up, with flashes of lightening.

8 A rainbow appears.

Think of the front of a shop which you often go to. Consider the picture that comes before your mind’s eye.

9 The overall appearance of the shop from the opposite side of the road.

10 A window display including colors, shape, and details of individual items for sale.

11 You are near the entrance. The color, shape, and details of the door.

12 You enter the shop and go to the counter. The counter assistant serves you. Money changes hands.

Finally, think of a country scene which involves trees, mountains, and a lake. Consider the picture that comes before your mind’s eye.

13 The contours of the landscape.

14 The color and shape of the trees.

15 The color and shape of the lake.

16 A strong wind blows on the tree and on the lake causing waves.
