# Supplementary material for: Principal Components Analysis Relates Hippocampal Trisynaptic Circuit to Vividness of Visual Mental Imagery
Source: Brain Behav. 2026 Aug 30;16(9):e71666. doi: 10.1002/brb3.71666 (PMC13526228; doi:10.1002/brb3.71666)
Supplement: Supplementary file 4 [file BRB3-16-e71666-s005.docx]

**TABLE S2**

**PCA Statistics Tables**

| **Hippocampal Subfield Volumes Descriptive Statistics** | | | |
| --- | --- | --- | --- |
|  | Mean | Std. Deviation | Analysis N |
| left_hippocampal_tail | 480.4725 | 61.86032 | 53 |
| left_subiculum | 414.9859 | 41.50284 | 53 |
| left_CA1 | 627.1140 | 68.51451 | 53 |
| left_hippocampal-fissure | 150.2666 | 26.85241 | 53 |
| left_presubiculum | 287.1873 | 41.95162 | 53 |
| left_parasubiculum | 63.5086 | 13.39880 | 53 |
| left_molecular_layer_HP | 474.9847 | 51.45302 | 53 |
| left_GC-ML-DG | 278.4466 | 29.14599 | 53 |
| left_CA3 | 201.7224 | 29.83263 | 53 |
| left_CA4 | 241.4987 | 24.44499 | 53 |
| left_fimbria | 66.6651 | 24.99709 | 53 |
| left_HATA | 55.9013 | 7.56744 | 53 |
| right_Hippocampal_tail | 508.2553 | 68.76564 | 53 |
| right_subiculum | 419.0405 | 46.25375 | 53 |
| right_CA1 | 663.5868 | 60.36618 | 53 |
| right_hippocampal-fissure | 155.9103 | 25.35721 | 53 |
| right_presubiculum | 263.1809 | 46.78206 | 53 |
| right_parasubiculum | 56.4622 | 12.17395 | 53 |
| right_molecular_layer_HP | 496.6748 | 55.93688 | 53 |
| right_GC-ML-DG | 303.1474 | 27.93251 | 53 |
| right_CA3 | 233.9539 | 31.70470 | 53 |
| right_CA4 | 263.3886 | 23.02915 | 53 |
| right_fimbria | 51.5515 | 19.82780 | 53 |
| right_HATA | 56.7681 | 12.29638 | 53 |
| bilateral_hippocampal_tail | 988.7278 | 122.68706 | 53 |
| bilateral_subiculum | 834.0264 | 81.39446 | 53 |
| bilateral_CA1 | 1290.7009 | 120.56948 | 53 |
| bilateral_hippocampal-fissure | 306.1769 | 48.76151 | 53 |
| bilateral_presubiculum | 550.3682 | 84.70876 | 53 |
| bilateral_parasubiculum | 119.9708 | 22.00735 | 53 |
| bilateral_molecular_layer_HP | 971.6595 | 100.85934 | 53 |
| bilateral_GC-ML-DG | 581.5940 | 53.66267 | 53 |
| bilateral_CA3 | 435.6762 | 55.17785 | 53 |
| bilateral_CA4 | 504.8873 | 44.23844 | 53 |
| bilateral_fimbria | 118.2166 | 40.65704 | 53 |
| bilateral_HATA | 112.6694 | 15.55887 | 53 |
