# Supplementary material for: Principal Components Analysis Relates Hippocampal Trisynaptic Circuit to Vividness of Visual Mental Imagery
Source: Brain Behav. 2026 Aug 30;16(9):e71666. doi: 10.1002/brb3.71666 (PMC13526228; doi:10.1002/brb3.71666)
Supplement: Supplementary file 5 [file BRB3-16-e71666-s006.docx]

**TABLE S4**

**PCA Statistics Tables**

| **Rotated Component Matrix^a^** | | | | | | |
| --- | --- | --- | --- | --- | --- | --- |
|  | Component | | | | | |
|  | 1 | 2 | 3 | 4 | 5 | 6 |
| bilateral_CA3 | .924 |  |  |  |  |  |
| right_CA3 | .898 |  |  |  |  |  |
| bilateral_CA4 | .881 | .288 |  |  |  |  |
| right_CA4 | .862 |  |  |  |  |  |
| bilateral_GCMLDG | .840 | .309 | .316 |  |  |  |
| left_CA3 | .826 | .340 |  |  |  |  |
| right_GCMLDG | .826 | .258 | .262 |  |  |  |
| left_CA4 | .812 | .309 |  |  |  |  |
| left_GCMLDG | .778 | .330 | .339 |  |  |  |
| bilateral_CA1 | .775 | .291 | .302 |  |  | .259 |
| left_CA1 | .748 | .323 | .308 |  |  |  |
| right_CA1 | .739 |  | .270 | .268 |  | .304 |
| left_hippocampal _tail | .588 | .557 |  |  |  |  |
| bilateral_presubiculum |  | .862 |  |  | .341 |  |
| right_presubiculum |  | .837 |  |  | .318 |  |
| left_presubiculum |  | .816 |  |  | .337 |  |
| right_subiculum | .355 | .768 |  |  |  |  |
| bilateral_molecular_layer_HP | .498 | .738 |  |  |  |  |
| right_hippocampal_tail | .443 | .731 |  |  |  |  |
| bilateral_subiculum | .424 | .722 | .319 |  |  |  |
| left_molecular_layer_HP | .499 | .713 |  |  |  |  |
| right_molecular_layer_HP | .451 | .694 |  |  |  |  |
| bilateral_hippocampal_tail | .543 | .680 |  |  |  |  |
| left_subiculum | .455 | .595 | .383 |  |  |  |
| bilateral_fimbria |  |  | .895 |  |  |  |
| left_fimbria |  |  | .836 |  |  |  |
| right_fimbria |  |  | .767 |  |  |  |
| bilateral_hippocampal_  fissure |  |  |  | .959 |  |  |
| right_hippocampal_fissure |  |  |  | .897 |  |  |
| left_hippocampal_fissure |  |  |  | .886 |  |  |
| bilateral_parasubiculum |  | .285 |  |  | .922 |  |
| left_parasubiculum |  |  |  |  | .807 |  |
| right_parasubiculum |  | .277 |  |  | .729 |  |
| bilateral_HATA |  |  |  |  |  | .930 |
| right_HATA |  |  |  |  |  | .878 |
| left_HATA | .356 |  |  |  |  | .646 |
| Extraction Method: Principal Component Analysis.  Rotation Method: Varimax with Kaiser Normalization. | | | | | | |
| a. Rotation converged in 7 iterations. | | | | | | |
