# Supplementary material for: Principal Components Analysis Relates Hippocampal Trisynaptic Circuit to Vividness of Visual Mental Imagery
Source: Brain Behav. 2026 Aug 30;16(9):e71666. doi: 10.1002/brb3.71666 (PMC13526228; doi:10.1002/brb3.71666)
Supplement: Supplementary file 6 [file BRB3-16-e71666-s007.docx]

**TABLE S6**

**Mixed model ANOVA summary tables with Component scores as the dependent variable**

**Between subjects effects summary table**

| Source | Type III Sum of Squares | df | Mean Square | F | Sig. | Partial Eta Squared | Noncent. Parameter | Observed Power |
| --- | --- | --- | --- | --- | --- | --- | --- | --- |
| Intercept | 2.898 | 1 | 2.898 | 3.528 | .079 | .181 | 3.528 | .423 |
| Age Group | .637 | 1 | .637 | .775 | .392 | .046 | .775 | .132 |
| ACE Group | 2.918 | 1 | 2.918 | 3.552 | .078 | .182 | 3.552 | .425 |
| VVIQ Group | 8.250 | 1 | 8.250 | 10.044 | .006 | .386 | 10.044 | .845 |
| Error | 13.142 | 16 | .821 |  |  |  |  |  |
| a. Computed using alpha = .05 | | | | | | | | |
